# Nasal mucosal melanoma presenting as central type vertigo: a case report

**DOI:** 10.1186/1757-1626-2-7149

**Published:** 2009-05-05

**Authors:** Konstantinos Nellas, Iordanis Konstantinidis, Alexandros Zevgaridis, Athanasia Printza, Ioannis Efstratiou

**Affiliations:** 1 2nd Academic ORL Department, Papageorgiou HospitalThessalonikiGreece; 2 Pathology Department, Papageorgiou HospitalThessalonikiGreece

## Abstract

Nasal mucosal melanoma presents usually with epistaxis, nasal obstruction and facial pain. However melanoma tends to give distant metastases at an early stage, having rare clinical presentations.

We present a 74-year old female patient with symptoms of central type vertigo caused by brain metastases. Clinical assessment for the detection of the primary site revealed a nasal mucosal melanoma originating from the posterior end of the left inferior turbinate. The patient received a combination of radio and chemotherapy being in relatively good condition 8 months later. This is the first reported case of a nasal mucosal melanoma with vertigo as the first presenting symptom.

## Introduction

Mucosal melanomas arising from the nasal cavity and paranasal sinuses are rare and account for less than 1% of all melanomas and less than 4% of all sinonasal tumours [1]. The most frequent symptoms at first presentation are epistaxis and unilateral nasal obstruction with a range of 85-90% followed by facial pain and facial deformity usually in advanced cases [1]. Although the tumour has an aggressive biologic behaviour, distant metastasis is not frequent at the time of diagnosis accounting the 10% of nasal melanoma cases [2]. We present the first reported case in the literature of a nasal melanoma presenting as central type vertigo.

## Case presentation

**Figure 1.**
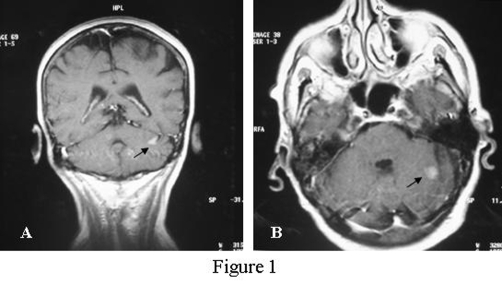
**(A)** Sagittal and **(B)**. Axial MRI T1 weighted sequence where it is noted (black arrows) a metastatic lesion on the left side of cerebellum.

**Figure 2.**
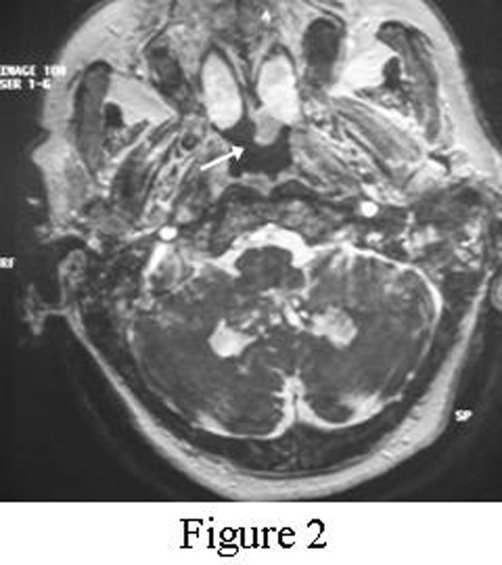
Axial MRI T2 weighted sequence in which a hypodense lesion is seen on the posterior end of the left inferior turbinate (white arrow).

**Figure 3.**
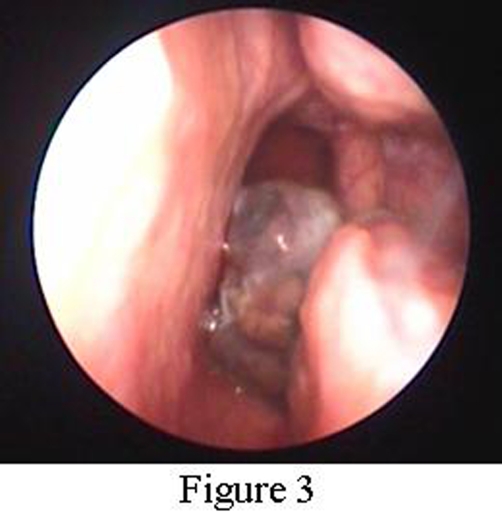
Endoscopic view of the left nasal cavity. A dark coloured mass is seen originating from the inferior turbinate obstructing partially the nasal choana.

**Figure 4.**
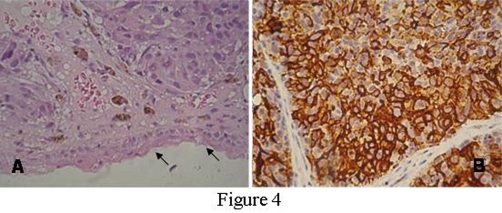
**(A)** Biopsy section showing nests of tumour cells with brownish melanin pigment within the submucosa of the nasal cavity. The arrowheads indicate the lining epithelia of the tumour mass (H&E × 400) **(B)** Immunohistochemistry positive stain of the tumor cells for HMB-45 marker × 400.

This is a case study of a non-smoker 74-year old female patient referred to ORL Department with a 2-months history of vertigo. History and clinical findings indicated the presence of vertigo with central type characteristics. Specifically the patient had a feeling of unsteadiness without spinning, absence of nausea and vomiting and a direction-changing nystagmus. Otologic assessment had normal findings and palpation of the neck did not reveal nodal disease. Magnetic resonance imaging (MRI) of the brain revealed 4 metastatic lesions. The location of metastases was the cerebellum (Figure 1) and temporal lobe on the left side and occipital and parietal lobe on the right side. Interestingly a small hypodense lesion in T2 weighted sequences was also revealed on the posterior end of the left inferior turbinate (Figure 2). Nasal endoscopy showed a dark coloured mass originating from the posterior end of the left inferior turbinate (Figure 3) and a biopsy followed under local anesthesia. The histopathological examination of the nasal lesion biopsy revealed pleomorphic melanin pigmented cells (Figure 4A). Immunohistochemistry showed that the tumour cells stained positively for S-100 and HMB-45 (Figure 4B), confirming the diagnosis of a malignant melanoma. Further workup, included an MRI of the neck, abdomen and chest, without evidence of other primary site or distant metastasis. Thorough dermatological examination did not reveal any site of primary cutaneous melanoma.

The presence of distant metastases and the absence of nasal symptoms such as epistaxis were contra-indications for surgical treatment. The patient followed a scheme of radiotherapy with 3000 cGy whole brain and primary site irradiation divided in 10 sessions followed by 3 cycles of chemotherapy with Dacarbazine (850 mg/m^2^). The patient 8 months after diagnosis still has no nasal complains however she developed lung metastatic lesions.

## Discussion

Nasal mucosal melanoma is a very rare malignancy with a high tendency to develop both regional and distant metastases. Amongst all melanomas, only 1.3% derives from the mucous membranes. Head and neck mucosal melanomas are approximately the half of them [1].

Both genders are affected equally, with the peak age incidence between fifth and eighth decade [1]. The usual clinical presentation of a nasal melanoma is this with nasal symptoms such as unilateral nasal obstruction and epistaxis (85-90% of cases) [1,2]. Other reported symptoms are facial pain, facial deformity, secretory otitis media, diplopia and dysphagia [3,4]. This is the first reported case to our knowledge of a nasal melanoma without local or regional symptoms and vertigo as the first presenting symptom.

If a balance disorder should be always investigated with MRI of the brain is still debatable. The use of MRI in the management of vertigo presents a low cost effectiveness [5]. However if a thorough clinical examination indicates a possible central nervous system (CNS) origin or reveal suspected neoplastic lesions then the use of an MRI to exclude among others and metastatic lesions is mandatory. In case of a CNS metastasis without nodal disease the differential diagnosis should focus on neoplasms with tendency for distant metastases such as mucosal melanomas of the head and neck.

The most frequent locations of sinonasal track melanomas are the lateral wall and turbinates (39%), followed by the septum (23%) and paranasal sinuses (22%) [4].

Nasal mucosa melanoma arises from the melanocytes that have migrated during embryologic development from neural crest to sinonasal mucosa. One third of these lesions are amelanotic. The rest are usually rich in melanin and appear pigmented (50-70%) [4].

Presence of melanin sets the pathologic diagnosis of mucosal melanoma. Since melanin is not always present, various markers are used to improve the accuracy of diagnosis. The immunohistochemichal markers positive for melanoma include vimentin (97.1%), S-100 protein (91%), tyrosinase (78%) and HMB-45 (76%) [6]. In other reports the HMB-45 marker is found to be even more sensitive (98%) [7].

Opposing to squamous cell carcinoma, mucosal melanoma tends to develop distant metastases more frequently to lungs and brain than cervical lymph nodes [8]. Sites of nasal mucosal melanoma distant metastasis according to their frequency are lung, liver, bone and brain [9].

The most fundamental treatment is wide resection of the primary tumour whenever possible. Radiotherapy combined with surgery is recommended in cases of local recurrence, distant metastases or incomplete lesion removal [9],

The role of systemic therapy either chemotherapy or immunotherapy still remains controversial [9]. Chemotherapy should be utilized as an adjuvant therapy as there is no evidence to be effective as the only modality of treatment [9].

The elder patients present treatment dilemmas regarding their prognosis, quality of life, risks of anaesthesia and surgery, and prolonged postoperative care. Decisions with respect to radical surgery in an elderly population must encompass quality of life issues in addition to that of the disease-free survival.

The prognosis of a nasal melanoma is poor, despite the progress in the imaging techniques. The mean survival for the mucosal melanoma at 5 years is reported 17.1%; with a range of 0-48% [4]. Independent prognostic factors appear to be the location, size, thickness, vascular invasion, positive lymph nodes and development of distant metastasis of the tumour [7,10]. Regarding the location some authors suggest that nasal melanomas of the septum have a better prognosis than that of the lateral wall of the nose [10].

The development of distant metastases from the time of diagnosis has a wide range (1-200 months) [1,2,8]. However the presence of distant disease is quickly followed by death for most with a median survival in several studies ranging from 3 to 6 months [1,2,4,9].

## Conclusion

In conclusion the presented case except its rarity provides information regarding the possible clinical presentation with symptoms from the central nervous system of an aggressive tumour such as the nasal mucosal melanoma.

## List of abbreviations

CNS: Central nervous system
MRI: Magnetic resonance imaging
ORL: Otorhinolaryngology

## References

[bib-001] Chang AE, Karnell LH, Menck HR (1998). The National Cancer Data Base report on cutaneous and non-cutaneous melanoma: a summary of 84,836 cases from the past decade. The American College of Surgeons Commission on Cancer and the American Cancer Society. Cancer.

[bib-002] Takagi M, Ishikawa G, Mori W (1974). Primary malignant melanoma of the oral cavity in Japan. With special reference to mucosal melanosis. Cancer.

[bib-003] Pandey M, Mathew A, Abraham EK, Ahamed IM, Nair KM (1998). Primary malignant melanoma of the mucous membranes. Eur J Surg Oncol.

[bib-004] Wagner M, Morris CG, Werning JW, Mendenhall WM (2008). Mucosal melanoma of the head and neck. Am J Clin Oncol.

[bib-005] Stewart MG, Chen AY, Wyatt JR, Favrot S, Beinart S, Coker NJ, Jenkins HA (1999). Cost- effectiveness of the diagnostic evaluation of vertigo. Laryngoscope.

[bib-006] Thompson LD, Wieneke JA, Miettinen M (2003). Sinonasal tract and nasopharyngeal melanomas: a clinicopathologic study of 115 cases with a proposed staging system. Am J Surg Pathol.

[bib-007] Dauer EH, Lewis JE, Rohlinger AL, Weaver AL, Olsen KD (2008). Sinonasal melanoma: a clinicopathologic review of 61 cases. Otolaryngol Head Neck Surg.

[bib-008] Rinaldo A, Shaha AR, Patel SG, Ferlito A (2001). Primary mucosal melanoma of the nasal cavity and paranasal sinuses. Acta Otolaryngol.

[bib-009] Huang SF, Liao CT, Kan CR, Chen IH (2007). Primary mucosal melanoma of the nasal cavity and paranasal sinuses: 12 years of experience. J Otolaryngol.

[bib-010] Bradley PJ (2006). Primary malignant mucosal melanoma of the head and neck. Curr Opin Otolaryngol Head Neck Surg.

